# West Nile Virus Infection

**DOI:** 10.3390/pathogens12020151

**Published:** 2023-01-17

**Authors:** Francisco Llorente

**Affiliations:** Centro de Investigación en Sanidad Animal (CISA-INIA), CSIC, Valdeolmos, 28130 Madrid, Spain; dgracia@inia.csic.es

West Nile virus (WNV) is a mosquito-borne pathogen that belongs to the *Flavivirus* genus (family *Flaviviridae*). The virus is maintained in nature in a rural cycle between mosquito vectors, mainly *Culex* species, and avian hosts. Spillover from this cycle occasionally results in outbreaks in horses and humans where, in severe cases, the infection can induce neurological signs such as meningitis and encephalitis, and in some cases can lead to death. The virus has spread in the world in this century, and an increase in the number of outbreaks as well as their severity has been observed in Europe in recent years. Considering all of these aspects, a multidisciplinary approach including public, animal, and environmental health, is the best option to increase the knowledge on this problem, making this disease an excellent example of a “One Health” issue.

In this Special Issue of *Pathogens*, readers can find contributions covering topics on different aspects of the virus, including its pathogenesis, vaccines, diagnosis and epidemiology, in both humans and animals. We have eight contributions in the form of original research and a review.

Six of the research papers published in this Special Issue are focused on epidemiological studies on different areas of the world including different continents where the epidemiological history of WNV has been completely different. In Africa, the virus has been present since its first description in 1937 in the West Nile district in Uganda and can now be considered as endemic in the continent. In Europe, WNV emerged in the 1960s, but only sporadic cases occurred in the continent up to the 1990s. Since then, the frequency and relevance of outbreaks have increased, reaching the highest number of cases in 2018 (2083 reported cases in the European Union). The presence of the virus in the Western Hemisphere is much more recent. The virus was detected for the first time in New York in 1999 and quickly spread across North America causing an epidemic with a high number of human cases and bird deaths. Between 2001 and 2004 it was present in the Caribbean, and in South America viral genome was detected in Argentina in 2006, Colombia in 2012 and Brazil in 2018.

Epidemiology in South Africa is the focus of the article from Bertram et al. [1] which describes the epidemiological situation and clinical presentation in horses during 2016–2017. Samples from horses that were positive to the disease were obtained by passive surveillance and compared with non-infected horses. The results obtained indicated that WNV cases were higher in young and non-vaccinated animals, but were also correlated with the season, high altitude, and the breed of the animals. The increase in cases observed in 2017 can be attributed to environmental factors favouring vector population. Data obtained indicate that annual vaccination is especially recommendable for animals younger than five years-old and highly purebred breeds, and that the best season for vaccine application is spring. A second study carried out in Africa in this Special Issue describes the detection of Koutango virus in sandflies in Niger for the first time [2]. Koutango is a divergent genetic variant that, depending on the authors, can be considered a different lineage of WNV, being the most distant lineage showing high genetic distance in relation to others, or a related virus species. Koutango has only been detected in Africa. Unlike other WNV lineages, it has mainly been found in rodents and ticks, and shows a higher virulence in mice than other previously assayed WNV strains. The virus detected in sandflies was isolated, sequenced, and inoculated in mice, causing 100% mortality. High pathogenicity observed in mice and a possible higher range of vectors as indicates the detection in sandflies, suggest that Koutango can be a relevant emerging pathogen.

Three other contributions reflect the epidemiological situation in recent years in different European regions. Beck et al. [3] report outbreaks in Southern France between 2015 and 2019 in humans, equids and wild birds. The isolation of WNV lineage 1 in 2015 in the Camargue region suggests an endemic situation in the area in the year with the most important outbreak in horses. In 2008, lineage 2 emerged producing the most important epidemics affecting humans in France. The increase of cases in the Camargue area in 2018 is correlated with an increase in *Culex pipiens* population. Additionally, this study also describes the presence of the virus in areas without previous WNV circulation with infections in humans, horses and wild birds. Additionally, the isolation of WNV lineage 2 from a raptor in 2018 demonstrated the emergence of lineage 2. A second original paper that focus in Europe describes a serosurveillance [4] in humans in the province of Siena in Italy from 2016 to 2019, in an area without human cases since 2017. Serum samples (1800) were analyzed by ELISA test, immunofluorescence assay, and virus-neutralization. Although a low prevalence, under 1%, was detected, active circulation was confirmed by the presence of IgM, showing, for the first time the circulation of the virus in the area. The authors indicate a trend inWNV expansion in Central Italy, and although no human cases have been detected in the area the application of preventive measures and surveillance is recommended. A third contribution related to the circulation of WNV in Europe presents a description of human clinical cases in the Czech Republic in 2018 [5]. Clinical, epidemiological and laboratory findings of five patients are described in detail. Four of the patients were confirmed for WNV infection and at least one of these was caused by lineage 2. The fifth patient seems to have been infected by Usutu virus (USUV), showing an antibodies titer against this virus that was much higher than that observed against WNV. The data indicates the simultaneous circulation of WNV lineage 2 and USUV in the country in 2018.

An example of the epidemiological situation in a South American country is presented by Costa et al. [6], showing new genetic evidence of WNV circulation in Brazil after the first genome detection in the country in 2018. Viral genome was detected and sequenced from three symptomatic horses from different Brazilian states. A phylogenetic analysis indicated that independent introduction events from North America were produced for the strains detected in this work and those previously described in 2018. The authors also summarize the past evidence of WNV circulation in Brazil in humans, horses and avian species, including serological detection and provide a climate-informed assessment of the transmission risk of WNV across the country. The previous and new data obtained indicate that both sporadic and endemic local transmission possibly explain the WNV epidemiology in the country.

In relation to WNV pathogenesis Gamino et al. [7] delved into the pathogenesis of WNV in birds. In a previous study the authors demonstrated the susceptibility of red-legged partridge (*Alectoris rufa*) to two Mediterranean WNV strains from Morocco and Spain via experimental infection, showing higher mortality and morbidity in the birds infected with the Moroccan isolate. In this study, differences in pathogenesis have been analysed to explain the different courses of infection and mortality. Although the virus was present in brain and showed a similar viral load, a more acute inflammatory reaction and necrosis were observed for the Moroccan strain. These data suggest that differences in neurovirulence between strains can be more significant than neuroinvasiveness in birds mortality, and that a higher virulence can be caused by a more acute and severe encephalitis.

In the area of diagnosis, Pérez-Ramírez et al. [8] present an evaluation of the capacity of veterinary labs in the Mediterranean and Black Sea regions for themolecular and serological detection of WNV by an external quality assessment (EQA). The study was performed in the context of a European Union funded project (MediLabSecure) whose objective is to create a framework to promote arbovirus surveillance under a One Health perspective. Before the EQA, a training program for WNV detection including workshops for molecular and serological diagnoses was implemented, and the learning as well as acquired capacity of the participants to incorporate the techniques into their own laboratories were determined in this study. Seventeen veterinary laboratories from 17 countries were evaluated. Differential molecular detection by real-time RT-PCR for WNV lineages 1 and 2 and USUV was highly satisfactory, mainly for WNV lineages while less than 50% of laboratories gave correct results (100%) by conventional RT-PCR for generic detection of flaviviruses. In relation to serological detection, results were excellent for the generic detection of WNV antibodies by competition ELISA, but some laboratories failed in the detection of IgM detection in samples with low titers. The evaluation carried out demonstrated that the training program was useful in upgrading the diagnostic capacities in veterinary laboratories of EU-neighboring countries.

In the last manuscript in this collection, Saiz [9] discusses in a review the situation of WNV vaccines in terms of their use in horses, birds and humans. The different barriers encountered to their development and possible commercialization are summarized, considering the fact that although different vaccines are commercially available for horses, none of them have been licensed for humans.

I hope that this Special Issue will contribute to further knowledge on WNV infection. As the Collection Editor, I would like to thank all of the authors, reviewers, and editorial personnel who have made this Issue a reality.
